# Chloral in the Pains of Parturition

**Published:** 1876-08

**Authors:** 


Chloral in the Pains of Parturition.—Dr. Polaillon stated, at the Paris Medical Society, that he had made trial of chloral in eighteen obstetrical cases, administering it as an enema in doses of thirty or forty-five grains dissolved in half an ounce of water or milk. According to the effect produced, or whether the whole was retained or not, a second enema was given at the end of an hour, and sometimes even a third. The quantity of chloral thus given varied from 30 to 120 grains; but the mean amount really absorbed did not exceed, on account of the rejection of a portion of the enemata, sixty or seventy-five grains. In all the cases it was given during the later hours of the period of dilatation, or during the expulsive period, and was habitually well borne. In some of the women the contractions were obviously less painful, without diminution of their frequency or energy, the labor terminating in the usual time; but in a somewhat larger number the uterine action was arrested, as well as the pains, the presentation remaining in the pelvis or at the vulva, so that in five cases out of the eighteen delivery had to be terminated with the forceps. In M. Polaillon's opinion, while chloral may be employed with advantage for assuaging excessive excitability of the uterus, or for the relief of pains produced by too violent contractions, it ought to be rejected in normal accouchements.—*Med. Times and Gaz.*, April 22, from *L'Union Med.*, April 15, 1876.

